# Post-perceptual processing during the attentional blink is modulated by inter-trial task expectancies

**DOI:** 10.3389/fnhum.2013.00627

**Published:** 2013-10-08

**Authors:** Jocelyn L. Sy, James C. Elliott, Barry Giesbrecht

**Affiliations:** ^1^Department of Psychology, Vanderbilt UniversityNashville, TN, USA; ^2^Department of Psychological and Brain Sciences, University of CaliforniaSanta Barbara, Santa Barbara, CA, USA; ^3^Institute for Collaborative Biotechnologies, University of CaliforniaSanta Barbara, Santa Barbara, CA, USA

**Keywords:** selective attention, event-related potentials, attentional blink, expectancy

## Abstract

The selective processing of goal-relevant information depends on an attention system that can flexibly adapt to changing task demands and expectations. Evidence from visual search tasks indicates that the perceptual selectivity of attention increases when the bottom-up demands of the task increase and when the expectations about task demands engendered by trial history are violated. Evidence from studies of the attentional blink (AB), which measures the temporal dynamics of attention, also indicates that perceptual selectivity during the AB is increased if the bottom-up task demands are increased. The present work tested whether expectations about task demands engendered by trial history also modulate perceptual selectivity during the AB. Two experiments tested the extent to which inter-trial switches in task demands reduced post-perceptual processing of targets presented during the AB. Experiment 1 indexed post-perceptual processing using the event-related potential (ERP) technique to isolate the context sensitive N400 ERP component evoked by words presented during the AB. Experiment 2 indexed post-perceptual processing using behavioral performance to determine the extent to which personal names survive the AB. The results of both experiments revealed that both electrophysiological (Exp. 1) and behavioral (Exp. 2) indices of post-perceptual processing were attenuated when consecutive trials differed in terms of their perceptual demands. The results are consistent with the notion that the selectivity of attention during the AB is modulated not only by within-trial task demands, but also can be flexibly determined by trial-by-trial expectations.

## INTRODUCTION

Human selective attention is often characterized as being flexible and dynamic, continually adapting to the information processing demands imposed by the external world and our internal goals and expectations (e.g., Corbetta and Shulman, 2002; Kastner and Pinsk, 2004; Vogel et al., 2005; Ristic and Giesbrecht, 2011; Franconeri et al., 2013). The flexibility of selective attention has been investigated by measuring the processing of stimuli that compete for attentional resources using behavioral or neuroimaging methods (e.g., Yantis and Johnston, 1990; Lavie and Tsal, 1994; Vogel et al., 2005). Demonstrations of the flexibility of attention come from studies showing that selective information processing is not fixed at either early or late stages of representation, but rather is sensitive to task demands. For instance, when attentional selectivity is measured by the behavioral interference caused by information presented at task-irrelevant spatial locations during visual search, both task demands and expectations influence the flexibility of attention. Specifically, increasing the bottom-up task demands by increasing the perceptual similarity between visual search targets and distractors can reduce the behavioral interference caused by task-irrelevant stimuli, suggesting that increasing the bottom-up task demands increases the perceptual selectivity of attention (e.g., Lavie and Cox, 1997). Other studies have demonstrated that the selectivity of attention during visual search is also modulated by expectations generated by trial-by-trial task dependencies. For example, during visual search tasks in which difficulty varies from trial-to-trial, when the difficulty on trial*_n_* and trial*_n_*_-__1_ are different (switch trials) the amount of interference caused by stimuli presented at task-irrelevant locations can be reduced compared to when the search difficulty on consecutive trials is the same (repeat trials, e.g., Theeuwes et al., 2004). Together these studies, as well as other similar behavioral and neuroimaging evidence (e.g., Yantis and Johnston, 1990; Handy et al., 2001; Yi et al., 2004), support the notion that the selectivity of spatial attention is not fixed, but rather flexibly adapts to both the inherent difficulty of the task as well as one’s expectations about the task.

The flexibility of attention has not only been observed in spatial visual search tasks, but also in studies designed to measure the temporal dynamics of attention. The temporal dynamics of attention are typically investigated by examining the influence of selecting and identifying one target (T1) on the processing of a subsequent target (T2). These targets can either be presented within a rapid sequence of distractors (e.g., Raymond et al., 1992; Chun and Potter, 1995) or presented briefly and then masked (e.g., Duncan et al., 1994; Ward et al., 1996). Observers typically have no difficulty reporting T1, but T2 detection and/or identification is impaired when it is presented within 200–500 ms of T1 (e.g., Raymond et al., 1992). This impairment is known as the attentional blink (AB) and it is thought to represent the temporal dynamics of selection and consolidation processes (for recent reviews, see Dux and Marois, 2009; Martens and Wyble, 2010). Classic behavioral and electrophysiological studies of the AB have demonstrated that despite the severe impairment in T2 performance, semantic information about T2 survives the AB and that items presented during the AB can prime subsequent targets (e.g., Luck et al., 1996; Maki et al., 1997; Shapiro et al., 1997; Vogel et al., 1998; Rolke et al., 2001; Dux and Marois, 2008). Based on this evidence, theoretical accounts of the AB typically assume that semantic processing is preserved during the AB and that the impairment in T2 performance occurs because of a post-perceptual failure of attention (e.g., Chun and Potter, 1995; Raymond et al., 1995; Olivers and Meeter, 2008).

In contrast to the studies showing spared semantic processing during the AB, more recent studies have demonstrated that semantic information about T2 does not always survive the AB. For instance, Vachon and Jolicoeur (2011) and Vachon et al. (2007) have reported both behavioral and electrophysiological evidence that semantic processing within the AB can be suppressed when there is a task-switch between T1 and T2. The reduction in semantic processing presumably occurs because the reconfiguration of the attentional-set from one task to the other is a resource-demanding process that interferes with the perceptual processing of T2 (Vachon et al., 2007; Vachon and Jolicoeur, 2011). Similarly, Giesbrecht et al. (2007, 2009) have used both electrophysiological and behavioral approaches to demonstrate that increasing T1 task load can suppress the extent to which semantic and high priority information (e.g., personal names) can survive the AB.

While the evidence from the AB showing reduced post-perceptual processing (i.e., increased selectivity) with increasing task demands parallels the results of the visual search tasks showing reduced flanker interference and increased perceptual selectivity with increased perceptual load, there is a critical difference: in the studies of the AB, the selectivity of attention is measured by post-perceptual processing of a task-relevant stimulus; whereas, in the visual search task selectivity is measured by the post-perceptual processing and subsequent interference caused by task-irrelevant stimuli. However, recent behavioral evidence has revealed that, much like in the visual search tasks described above, increasing T1-task load can reduce the interference caused by task-irrelevant flankers presented simultaneously with T2 during the AB (Elliott and Giesbrecht, 2010). Thus, when one considers the evidence together, the data are consistent with the notion that the perceptual demands of the T1 task can modulate the selectivity of attention within the AB, when it is measured by the post-perceptual processing of task-relevant information and when it is measured by the post-perceptual processing of task-irrelevant information.

The recent empirical evidence in the literature is consistent with the notion that the selectivity of attention during the AB is flexible and modulated by the T1 task demands. However, it is unclear whether the temporal dynamics of attention are modulated by expectancies generated by inter-trial dependencies of T1 task demands. To clarify this issue, we tested whether the expectancies engendered by task-demand dependencies between trials modulate post-perceptual processing during the AB. In two experiments, participants were presented with two masked targets displayed in rapid succession. In both experiments, the first target (T1) was a flanker-type stimulus consisting of a single arrow flanked by pairs of arrows pointing either in the same direction (congruent, e.g., >>>>>) or in different directions (incongruent, e.g., <<><<). We refer to the congruent and incongruent conditions as low and high T1 load, respectively (Giesbrecht et al., 2007, 2009). Unlike previous studies that have used blocked T1 load conditions to demonstrate the effects load on post-perceptual processing of information presented during the AB (i.e., Giesbrecht et al., 2007, 2009), in the present experiment the two types of T1 load trials were randomly intermixed within experimental blocks. The random intermixing of trials allowed us to investigate the effects of inter-trial dependencies on post-perceptual processing during the AB by permitting the analysis of the data as a function of whether the T1-load on a given trial was the same as the previous trial (i.e., a T1-repeat trial) or was different than the previous trial (i.e., a T1-switch trial). In Experiment 1, post-perceptual processing during the AB was indexed by measuring the context sensitive N400 event-related potential (ERP) evoked by T2. In Experiment 2, post-perceptual processing was indexed by measuring the extent to which personal names survive the AB. Based on studies of spatial attention (Theeuwes et al., 2004) and previous studies of the AB (Giesbrecht et al., 2007, 2009; Vachon et al., 2007; Elliott and Giesbrecht, 2010; Vachon and Jolicoeur, 2011), we predicted that the additional demands required on T1-switch trials should decrease post-perceptual processing during the AB, relative to T1-repeat trials. Consistent with this prediction, we observed that T1-switches in load resulted in less semantic processing during the AB in both experiments.

## EXPERIMENT 1

### RATIONALE

The purpose of Experiment 1 was to test if expectancies generated by inter-trial T1 task dependencies modulate the processing and availability of semantic information presented during the AB. To do so, we revisited the finding that the context-sensitive N400 ERP component survives the AB (Luck et al., 1996). A context word was presented at the beginning of each trial, followed by a masked flanker stimulus (T1) and a word (T2) that was either related or unrelated to the context word presented at the beginning of the trial. The magnitude of the context sensitive N400 ERP (e.g., Kutas and Hillyard, 1980) was quantified by computing the mean amplitude of the difference wave of unrelated–related trials between 300 and 500 ms post T2 stimulus onset (Luck et al., 1996; Vogel et al., 1998). Using a similar task in which high and low T1 load were presented in different blocks of trials, we previously demonstrated that the N400 evoked by T2 was not modulated by the AB when T1 load was low, but was completely suppressed during the AB when T1 load was high (Giesbrecht et al., 2007). The key issue in the present work is whether trial-by-trial dependencies generated when T1 load is mixed within a block of trials alters this pattern. Specifically, if semantic processing of T2 is not constrained by expectancies engendered by inter-trial T1 task dependencies, then an N400 should be observed in all conditions. However, if the attentional demand imposed by inter-trial T1-switches modulates the extent to which semantic processing occurs, then the magnitude of the N400 should be reduced during the AB under switch compared to repeated conditions.

### MATERIALS AND METHODS

#### Participants

Twelve undergraduates from the University of California, Santa Barbara (UCSB) provided informed consent and were paid $10/hour for their participation (mean age = 19; 9 female). The UCSB Human Subjects Committee approved all procedures.

#### Apparatus and stimuli

Stimulus presentation was controlled using custom scripts written for MATLAB (Mathworks, Inc., Boston, MA, USA) and the Psychophysics Toolbox (Brainard, 1997). T1 stimuli were black and consisted of a central arrow (0.4° × 0.4°) centered between two pairs of arrows (0.4° × 1.1°). The distance between adjacent arrows was 0.15°. The complete target stimulus subtended 0.4° × 2.6°. The context word presented at the beginning of the trial and the T2 word were black and white, respectively. Both were presented in uppercase 32-point Arial font. Each character subtended approximately 0.4° × 0.4°. T1 and T2 masks were strings of black numbers and uppercase letters the same length as the respective target. All stimuli were presented on a neutral gray background and viewed on a 19-inch color monitor from a distance of 125 cm.

#### Procedure

**FIGURE 1:**
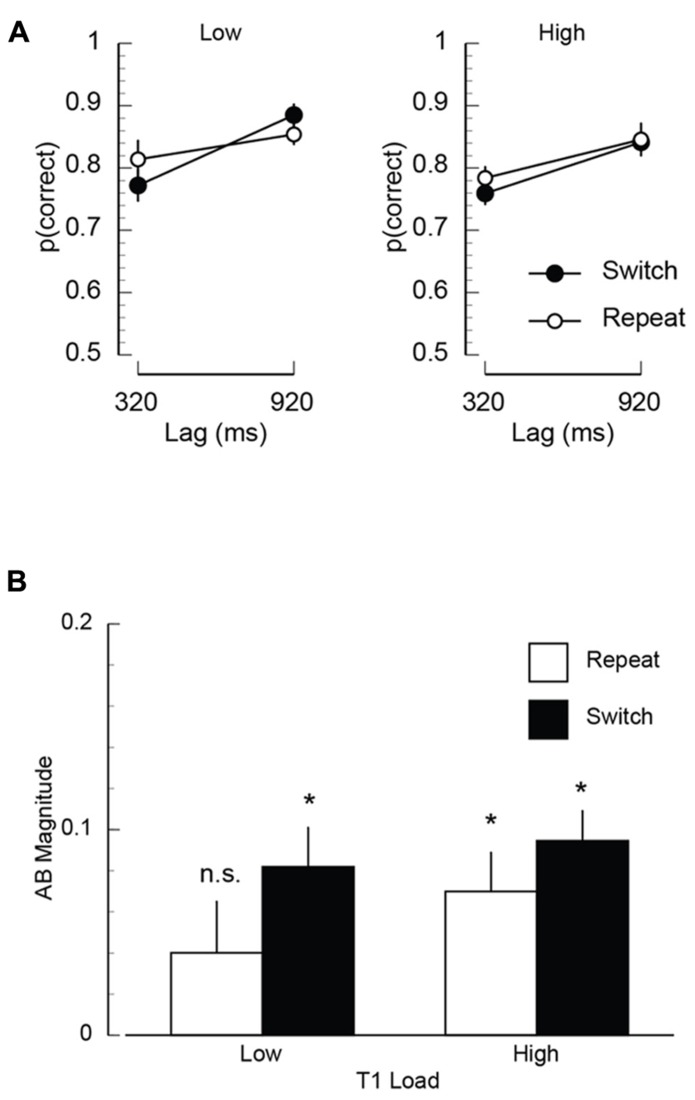
**Mean task 2 behavioral performance in Experiment 1.** **(A)** Mean proportion of correct responses on the second target (T2) task, plotted as a function of T1 load (high/low), T1–T2 lag (320/920 ms), and inter-trial T1-load dependency (repeat/switch). **(B)** Mean AB magnitude plotted as a function of T1 load and inter-trial T1-load dependency. Asterisks indicate that AB magnitude was significantly different than zero at *p* < 0.05, FDR-corrected for multiple comparisons.

Each trial began with a random fixation interval (500–1000 ms), followed by the context word (1000 ms). After the context word there was a second random delay (750–1250 ms), followed by the presentation of T1 (53.3 ms) and the T1 mask (53.3 ms; T1-mask ISI = 53.3 ms). After the temporal lag (either 320 or 920 ms) lapsed, T2 was presented (40 ms) and then masked (40 ms; T2-mask ISI = 40 ms). After a third random delay (750–1250 ms) subjects were prompted to indicate their responses for T1 and T2. Subjects were instructed to read the context word presented at the beginning of the trial, identify the direction of the T1 central arrow (left or right) and determine whether T2 was related or unrelated to the context word. All responses were unspeeded and typed into the keyboard. After the responses were recorded, fixation returned to the screen and the participant started the next trial when ready. A sample trial sequence is shown in **Figure 1**.

**FIGURE 2:**
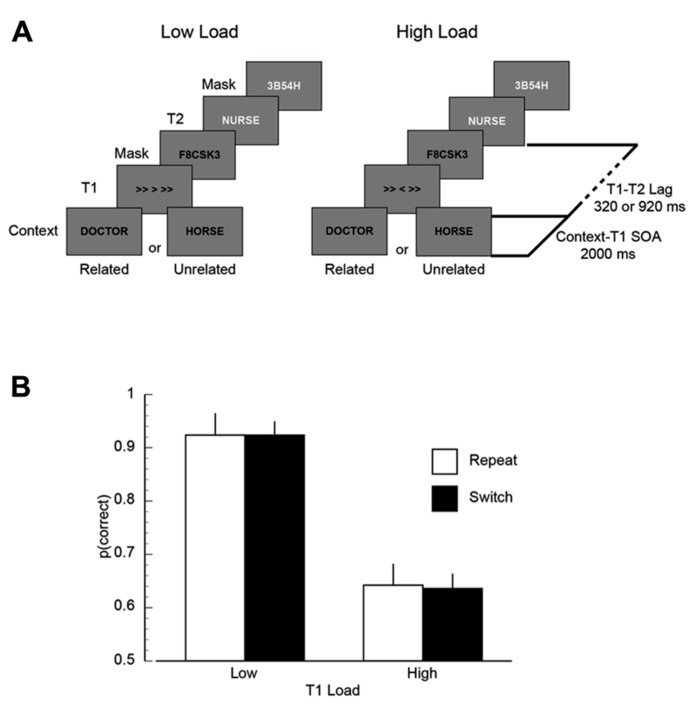
**(A)** A schematic illustration of the trial sequence in Experiment 1. **(B)** Mean proportion of correct responses on the first target (T1) task, plotted as a function of T1 load (high/low) and inter-trial T1-load dependency (repeat/switch). In this and subsequent figures, error bars represent the standard error of the mean calculated in a manner appropriate for within subjects experimental designs (Loftus and Masson, 1994).

#### Design

There were four independent variables: T1 load, T1 inter-trial dependency, T2-relationship, and T1–T2 lag. T1 load was manipulated by the direction of the flankers relative to the central arrow and was either congruent (i.e., >>>>> or <<<<<) or incongruent (i.e., <<><< or >><>>). Because the different T1 load conditions were intermixed, each trial could be categorized as T1-repeat trial (when T1-load on trial*_n_* was the same as trial*_n_*_-__1_) or T1-switch trial (when T1-load on trial*_n_*, was different from trial*_n_*_-__1_). T2-relationship specified the semantic association, either related or unrelated, between T2 and the context word. The specific words were compiled from previously published studies and norms (Postman and Keppel, 1970; Giesbrecht et al., 2004, 2007) and consisted of 300 related word pairs. Each word pair was randomly assigned to each of the load conditions, under the constraint that across subjects each pair was assigned to each of the load conditions an equal number of times. Unrelated word lists were created by randomly shuffling the related word pairs (Giesbrecht and Kingstone, 2004; Smallwood et al., 2011). T1–T2 lag was the temporal interval between the onsets of T1 and T2 and it was either 320 or 920 ms. T2-relationship and T1–T2 lag conditions were randomly intermixed within each block. There were 600 total trials (75 trials in each condition) that were divided into 10 blocks (five for each load condition) of 60 trials. Prior to the experimental trials, subjects were given 10 practice trials.

#### Recording and analysis

Electroencephalographic (EEG) activity was recorded at 256 Hz from 32 Ag/AgCl sintered electrodes mounted in an elastic cap and placed according to the International 10/20 System. The horizontal and vertical electrooculograms (EOG) were recorded from electrodes placed 1 cm lateral to the external canthi (left and right) and above and below each eye, respectively. The data were re-referenced offline to the average of the signal recorded from electrodes placed on the left and right mastoids and then band-pass filtered (0.1–30 Hz). Trials containing ocular artifacts (blinks and eye movements) detected by EOG amplitudes exceeding ± 50 mV or by visual inspection were excluded from the analysis. The average percentage of trials that were rejected was 6.9% (range 1.3–15.2%).

The average ERP waveforms in all conditions were computed time-locked to the onset of T2 and included a 200 ms prestimulus baseline and 600 ms poststimulus interval. The N400 was isolated by subtracting the resulting ERP waveforms on related trials from the ERP waveforms on unrelated trials. It is important to note that for a given subject, lag, and load condition the T2 word was exactly the same (only the context word was different), therefore any modulations observed in the resulting difference wave cannot be attributed to physical stimulus differences. The magnitude of the N400 was quantified as the mean amplitude of the difference waves over the 300–500 ms post-T2 time window. N400 measurements were obtained from frontal, central, and parietal electrodes (F3, Fz, F4, C3, Cz, C4, P3, Pz, P4, Luck et al., 1996; Vogel et al., 1998; Giesbrecht et al., 2007). As with previous studies, the mean amplitudes included both T2 correct and T2 incorrect trials (Luck et al., 1996; Vogel et al., 1998; Giesbrecht et al., 2007). The inclusion of both correct and incorrect trials should increase the likelihood that an N400 will be observed during the AB because semantic access is more likely to occur on T2 correct trials. Thus, any observed reduction in the magnitude of the N400 during the AB is likely to be an underestimate of the true reduction of semantic processing. Unless mentioned otherwise, within-subjects ANOVAs were used for all statistical analyses, and the *p*-values were adjusted in accordance with the Greenhouse-Geisser epsilon value.

### RESULTS

#### Behavior

*T1 accuracy*. Mean proportion of correct T1 responses are plotted as a function of T1 load (low/high) and inter-trial dependency (repeat/switch) in **Figure 1B**. Overall mean performance was 0.78 (SEM = 0.035). There was a significant effect of T1 load, such that performance was lower when T1 load was high (*M* = 0.64, SEM = 0.068) relative to when T1 load was low (*M* = 0.92, SEM = 0.021; *F*(1,11) = 15.58, *p* < 0.003, MSE = 0.062). Neither the main effect of inter-trial dependency nor the load x inter-trial dependency interaction were significant (both *F*’s < 1).

*T2 accuracy*. Mean proportion of correct T2 responses are plotted as a function of T1 load, inter-trial dependency, and lag in **Figure 2A**. Overall performance was lower at short lags compared to long lags (*F*(1,11) = 9.81, *p* < 0.02, MSE = 0.013). While visual inspection of **Figure 2A** suggests that there is an interaction between inter-trial dependency and lag, such that at the short lags performance on switch trials was lower than repeat trials, this interaction was not significant (*F*(1,11) = 2.16, *p* = 0.17, MSE = 0.013). No other effects were statistically significant.

*AB magnitude*. Two analyses were performed using AB magnitude as an index of the severity of the performance decrement caused by the T1 load and trial dependency manipulations. AB magnitude was computed by subtracting each individual’s performance at the short lag (320 ms) from an optimal performance baseline (Jackson and Raymond, 2006; Giesbrecht et al., 2009). In the present experiment, the performance baseline for all conditions was the accuracy in the 920 ms lag, low load-repeat condition (i.e., the condition in which T2 accuracy should be optimal). It was appropriate to select this data point to serve as the optimal performance baseline for all conditions because the T2 stimuli were exactly the same in all conditions. It is important to note that because AB magnitude was computed relative to a single estimate of optimal performance (i.e., 920 ms lag, low load-repeat condition) instead of relative to a within condition estimate of optimal performance (e.g., the 920 ms lag within each condition), the ANOVA on AB magnitude was not redundant with the ANOVA on T2 accuracy including lag as a factor reported in the preceding paragraph. Using this metric of AB magnitude, the first analysis tested whether the severity of the AB was modulated by trial dependency and load using a repeated measures ANOVA. The results of this analysis revealed a trend for an effect of inter-trial dependency (*F*(1,11) = 3.40, *p* < 0.1), but no other significant effects. While the ANOVA using this metric of AB magnitude as the dependent measure can indicate whether the severity of the AB is modulated by the experimental factors, it does not indicate the presence of an AB within a specific condition. Thus, the second analysis tested for the presence of an AB within each condition. To identify the presence of the AB, one-sample *t*-tests were performed, testing whether the AB magnitude in each condition was significantly different than zero (i.e., no AB). A false discovery rate correction (FDR; Benjamin and Hochberg, 1995) was applied to correct for multiple comparisons (*p* < 0.05). The results of this analysis are shown in **Figure 2B**. The key finding of this analysis was that AB magnitude was significantly different than zero in all conditions (FDR-corrected *p*’s < 0.006), except for the repeat low load condition (FDR-corrected *p* > 0.28).

#### Electrophysiology

**FIGURE 3:**
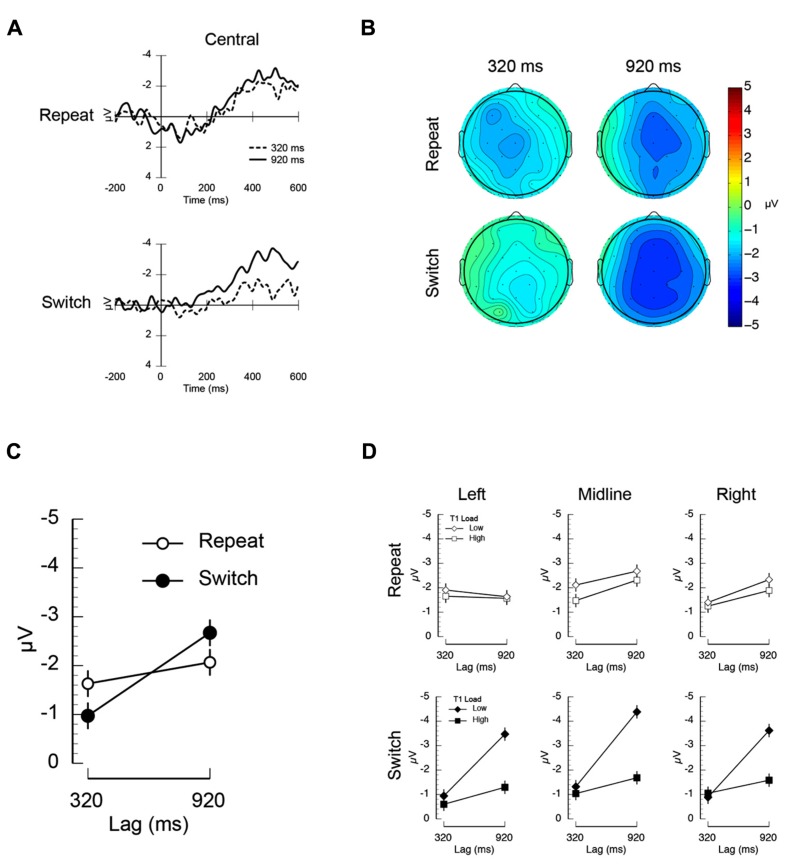
**Electrophysiological results from Experiment 2.** **(A)** Mean unrelated-related differences waves illustrating the N400 measured at central electrodes (average at electrodes C3/Cz/C4). **(B)** Scalp topography of the N400 mean amplitude computed over the 300–500 ms time window. **(C)** Mean N400 amplitude plotted as a function of lag (320/920 ms) and inter-trial dependency. The mean amplitude was computed over the 300–500 ms post-T2 time window and averaged across all electrode sites included in the analysis (see Materials and Methods). **(D)** Mean N400 amplitude measured at left (F3/C3/P3), midline (Fz/Cz/Pz), and right electrodes (F4/C4/P4) plotted as a function of inter-trial dependency (repeat/switch), lag (320/920 ms), and T1 load (low/high).

The ERP results are summarized in **Figure 3**. The mean N400 difference waves measured at central electrodes (C3/Cz/C4) are shown in **Figure 3A** as a function of inter-trial dependency, lag and time. The scalp topography during the N400 time window is shown in **Figure 3B**. The mean amplitude at all electrodes included in the analysis is plotted as a function of inter-trial dependency and lag in **Figure 3C**. Finally, the N400 mean amplitude is plotted as a function of inter-trial dependency, load, and lag for left, midline, and right electrodes in **Figure 3D**. The mean amplitudes were entered into a repeated measures ANOVA that included T1-load, inter-trial dependency, lag, anterior-posterior electrode position (frontal, central, parietal), and left-right electrode position (left, midline, right) as factors. The key finding that emerged from this analysis was a significant interaction between inter-trial dependency and lag (*F*(1,11) = 5.29, *p* < 0.05, MSE = 16.32). Inspection of **Figure 3C** suggests that this interaction is being driven by the fact that the N400 is not modulated by lag on repeat trials, but is on switch trials. *Post-hoc* *t*-tests confirmed this interpretation by revealing that there was no effect of lag on T1-repeat trials (*t*(11) = 1.07, *p* > 0.30), but the N400 was significantly smaller at the 320 ms lag than the 920 ms lag (*t*(11) = 2.93, *p* < 0.02) on T1-switch trials. This interaction is clearly visible not only in the mean amplitude data (**Figure 3C**), but also in the waveforms and scalp topographies (**Figures 3A,B**), all of which show a robust N400 on T1-repeat trials both inside and outside the AB, but a reduced N400 on T1-switch trials during the AB. There was also a three-way interaction between inter-trial dependency, lag, and electrode left-right position (*F*(2,22) = 5.34, *p* < 0.014, MSE = 0.788). This interaction (plotted with the additional factor of load in **Figure 3D**), was such that the inter-trial dependency × lag interaction (i.e., an effect of lag on switch trials, but not on repeat trials) was stronger at left electrode sites than midline and right electrode sites. Interestingly, while there is suggestive visual evidence that the effect of switching from high to low load had a greater impact on the N400 at short temporal lags than switching from low to high load, the three-way interaction was not significant (*F*(1,11) = 2.92, *p* > 0.12, MSE = 22.51). The remaining main effects and interactions were also not statistically significant.

**FIGURE 4:**
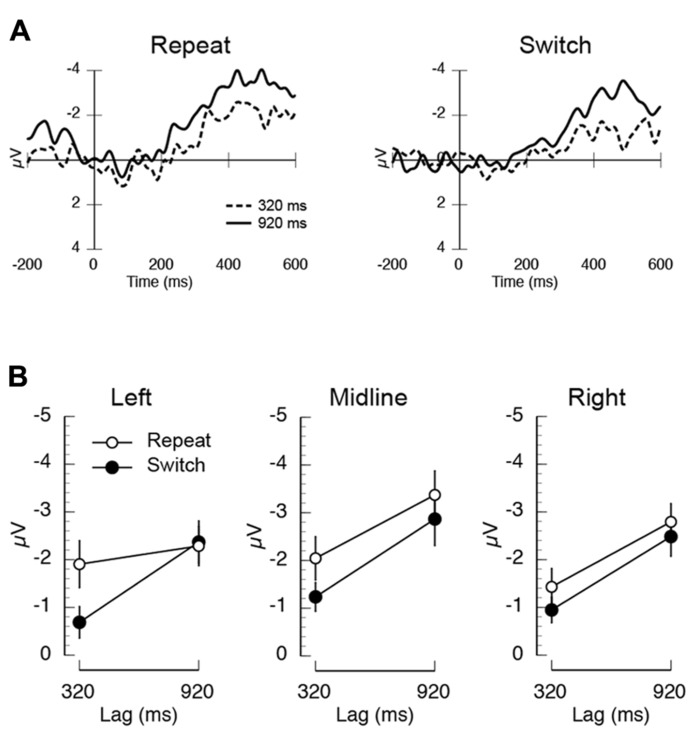
**Results of the control analysis of the electrophysiological data from Experiment 2 using a 50 ms prestimulus baseline.** **(A)** Mean unrelated-related differences waves illustrating the N400 measured at central electrodes (average at electrodes C3/Cz/C4). **(B)** Mean N400 amplitude measured at left (F3/C3/P3), midline (Fz/Cz/Pz), and right electrodes (F4/C4/P4) plotted as a function of inter-trial dependency (repeat/switch) and lag (320/920 ms).

Visual inspection of the difference ERP waveforms plotted in **Figure 3A** suggests that the baseline of the 920 ms lag waveform on repeat trials is generally more positive than the corresponding condition on switch trials. To assess the extent to which this apparent modulation in the baseline is contributing to the inter-trial dependency × lag interaction, we ran a control analysis using a finer-grained pre-stimulus interval (50 ms). The resulting rebaselined difference waves and mean amplitudes are shown in **Figure 4**. While the overall inter-trial dependency × lag interaction failed to reach significance, the inter-trial dependency × lag × electrode left-right position was significant (*F*(2,22) = 4.06, *p* < 0.04, MSE = 1.69). As in the original analysis, and as can be clearly observed in the mean amplitudes shown in **Figure 4B**, this interaction was such that the inter-trial dependency × lag interaction was robust over left electrodes. In contrast, at midline and right electrodes, the primary modulator of the N400 was temporal lag. This control analysis suggests that the inter-trial dependency × lag interaction is not solely being driven by differences in the prestimulus baseline, but rather is being driven by changes that are mediated by the interaction between trial-by-trial expectancies about task demands and the attentional demands caused by the AB.

### SUMMARY

The key finding in Experiment 1 was that the magnitude of the N400 was attenuated during the AB on T1-switch trials, but not on T1-repeat trials. This finding suggests that post-perceptual processing during the AB was modulated by the inter-trial task-demand expectancies and that the violation of this expectancy on T1-switch trials served to increase the selectivity of attention compared to when this expectancy was not violated. Interestingly, while there was an inter-trial dependency × lag interaction there was not an interaction between dependency, load, and lag. In other words, the dependency × lag interaction described above, was not modulated by load. This is interesting because it suggests that the previously reported effect of load on the N400, which serves to completely suppress the N400 during the AB (Giesbrecht et al., 2007), can be reversed by the context provided by the inter-trial dependencies.

A second result was that while inter-trial dependency did not influence T1 accuracy or overall T2 accuracy, there was suggestive evidence that dependency did affect the presence of the AB. Specifically, there was a significant AB on T1-low load switch trials, but not on T1-low load repeat trials. Interestingly, in our previous study, an AB was observed even when T1-load was low (Giesbrecht et al., 2007). The absence of a significant AB in the low-load repeat condition suggests that the expectancy generated by the inter-trial dependency causes a decrease in the difficulty of the T1 task that is sufficient to result in the absence of the AB on low-load trials. However, this result should be interpreted with caution because of the lack of an effect of dependency on T1 accuracy, the lack of an interaction between dependency and lag on T2 accuracy, and the lack of a significant main effect of trial dependency on AB magnitude.

## EXPERIMENT 2

### RATIONALE

To provide additional evidence that expectations engendered by trial-by-trial dependencies can modulate the selectivity of attention during the AB, we revisited another classic demonstration of post-perceptual processing during the AB: the finding that one’s own name is not subject to the AB (Shapiro et al., 1997). Experiment 2 tested whether the extent to which one’s own name survives the AB is modulated by inter-trial load. There were two key manipulations. First, both T1-load and inter-trial dependency were manipulated utilizing the same flanker task as in Experiment 1. However, because the behavioral effects on T1 performance and T2 performance were weak, we changed the T1 stimulus from black to white. The rationale was that the color change would make the flankers more salient and increase the likelihood that they would interfere with performance. Second, T2 was either the participant’s own name (T2-own) or someone else’s name (T2-other). If processing of high priority information during the AB is not constrained by task demands imposed by a switch trial, then there should be no AB for T2-own, but there should be an AB for T2-other, irrespective of switch in T1 congruency. However, if switches between trials influence the extent to which high priority information is processed, then the difference in AB magnitude between T2-own and T2-other conditions should be attenuated on switch trials compared to repeat trials.

### MATERIALS AND METHODS

#### Participants

Fifteen undergraduates from the University of California, Santa Barbara participated in a single 45 min session for credit in an introductory psychology class (8 female).

#### Equipment and stimuli

The T1 and mask stimuli, equipment, and stimulus control procedures were the same as in Experiment 1. The T2 stimuli were the subject’s own name and names from the database of registered birth names available from the United States Social Security Administration (). To provide a rough control for exposure to names other than one’s own name, the 50 most popular male and female names were selected from the list of names that corresponded to the most common year of birth of the largely freshman introductory psychology class from which our sample was drawn (1987). All names were presented in black uppercase 32 point Arial font. Each character subtended 0.4° × 0.4°.

#### Design

There were two changes in the design from Experiment 1. First, T2 was either the participant’s own name or another name from the list. The participant’s own name appeared on one eighth of the trials. The lag between the onsets of the first and second targets ranged from 200 to 800 ms in steps of 120 ms. All variables were combined factorially and randomly intermixed.

#### Procedure

**FIGURE 5:**
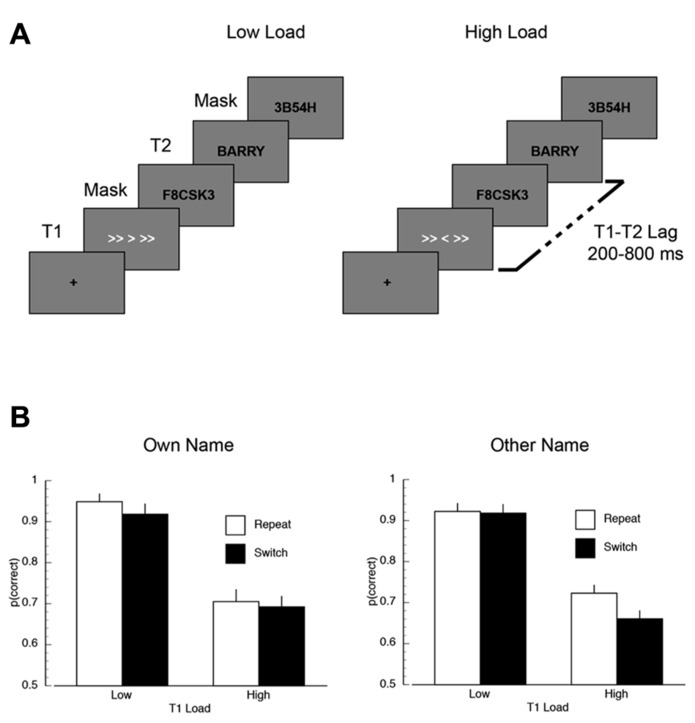
**(A)** A schematic illustration of the trial sequence in Experiment 2. **(B)** Mean proportion of correct responses on the first target (T1) task, plotted as a function of T2 name (own/other), T1 load (high/low), and inter-trial T1-load dependency (repeat/switch).

Each trial started with a fixation cross that remained on the screen until the participant initiated the trial by pressing the space bar. After the trial was initiated, there was a random delay (500–1000 ms) followed by the presentation of T1 and its mask (duration = 53.3 ms; T1-mask ISI = 53.3 ms). After the lapsing of the temporal lag, T2 was presented (40 ms) and then masked (40 ms; T2-mask ISI = 40 ms). On half the trials T2 was a male name and on the other half it was a female name. At the end of the trial, participants were instructed to indicate the direction of the central arrow (left or right) and then whether the name was a male or a female name. All responses were unspeeded and typed into the keyboard. After the responses were indicated, the fixation cross reappeared, and the participant started the next trial when ready. An example of the trial sequence is shown in **Figure 5A**. Participants completed one block of 10 practice trials, followed by 10 blocks of 48 trials.

### RESULTS

*T1 task accuracy.* The mean proportion of correct responses is plotted as a function of inter-trial dependency, load, and name in **Figure 5B**. There was a significant main effect of inter-trial switch where T1 accuracy was worse in switch trials (*M* = 0.80) compared to repeat trials (*M* = 0.83; *F*(1,14) = 10.23, *p* < 0.007, MSE = 0.002). Overall performance was also higher on low load trials (*M* = 0.93) than on high load trials (*M* = 0.70; *F*(1,14) = 33.34, *p* < 0.001, MSE = 0.048). The only significant interaction was the inter-trial dependency × load × name interaction (*F*(1,14) = 4.87, *p* < 0.05, MSE = 0.002), which appeared to be driven by overall lower performance in the T1-switch high load condition when T2 was someone else’s name.

**FIGURE 6:**
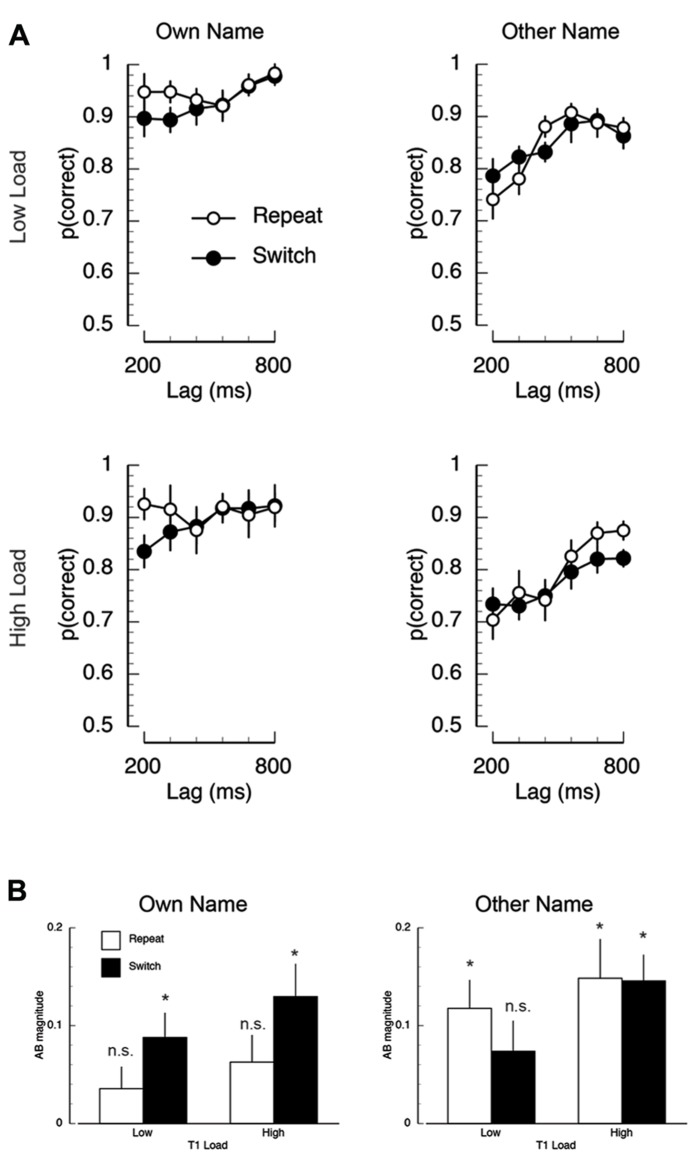
**Mean task 2 behavioral performance in Experiment 2.** **(A)** Mean proportion of correct responses on the second target (T2) task, plotted as a function of T1 load (high/low), T2-name (own/other), T1–T2 lag (320/920 ms), and inter-trial T1-load dependency (repeat/switch). **(B)** Mean AB magnitude plotted as a function of T2-name (own/other), T1 load, and inter-trial T1-load dependency. Asterisks indicate that AB magnitude was significantly different than zero at *p* < 0.05, FDR-corrected for multiple comparisons.

*T2 task accuracy.* The mean proportion of correct T2 responses is shown in **Figure 6A**. Overall, inter-trial dependency modulated performance, such that overall performance was lower on switch trials than on repeat trials (*F*(1,14) = 5.53, *p* < 0.04, MSE = 0.007). Mean accuracy was also lower for T2-other (*M* = 0.82) compared to T2-own (*M* = 0.92; *F*(1,14) = 54.84, *p* < 0.001, MSE = 0.035). There was a main effect of lag, where T2 report was worse at shorter lags than longer lags (*F*(5,70) = 11.36, *p* < 0.001, MSE = 0.013).

There were two key interactions. First, the effect of lag was more severe for T2-other compared to T2-own (*F*(5,70) = 2.45, *p* < 0.05, MSE = 0.017). Second, and most critically, there was a three-way interaction between inter-trial dependency, name, and lag (*F*(5,70) = 2.46, *p* < 0.05, MSE = 0.010). *Post-hoc* repeated measures ANOVAs revealed that this interaction was driven by the modulation of the name × lag interaction as a function of task dependency. Specifically, on repeat trials, there was no effect of lag for T2-own, but a large effect of lag for T2-other (name × trial: *F*(5,70) = 3.64, *p* < 0.006, MSE = 0.018). In contrast, on switch trials, there was an effect of lag (*F*(1,14) = 42.47, *p* < 0.001, MSE = 0.02) and name (*F*(1,14) = 17.60, *p* < 0.002, MSE = 0.015), but no interaction (*F* < 1).

*AB magnitude. *To further address the influence of inter-trial task dependencies on post-perceptual processing, we performed two AB magnitude analyses similar to those performed in Experiment 1. AB magnitude was computed by subtracting mean performance during the AB (lags 200–320 ms) from an optimal performance baseline. The baseline used in Experiment 2 was the condition in which T1-load was repeated and in which T2 was presented at the longest lag (800 ms). The resulting mean AB magnitude data are shown in **Figure 6B**. In the first analysis, AB magnitude was entered into a repeated measures ANOVA. The key finding was that AB magnitude was modulated by the interaction between inter-trial dependency and name (*F*(1,14) = 6.73, *p* < 0.022, MSE = 0.043). *Post-hoc* tests revealed two interesting aspects to this interaction. First, in the T2-own condition, AB magnitude was significantly smaller on repeat trials than on switch trials (*M*_T2-own, repeat_ = 0.05, *M*_T2-own, switch_ = 0.109; *t*(14) = 3.04, *p* < 0.009). In contrast, in the T2-other condition there was no difference between repeat and switch trials (*M*_T2-other, repeat_ = 0.13, *M*_T2-other, switch_ = 0.11; *t*(14) = 1.25, *p* > 0.23). Second, on repeat trials AB magnitude was significantly larger in the T2-other condition compared to the T2-own condition (*M*_T2-other, repeat_ = 0.05, *M*_T2-own, repeat_ = 0.13; *t*(14) = 2.74, *p* < 0.02), but on switch trials there was no difference in AB magnitude (*M*_T2-other, switch_ = 0.11, *M*_T2-own, switch_ = 0.109; *t*(14) = 0.04, *p* < 0.97). In the second analysis, just as in Experiment 1, the presence of the AB in each condition was identified using one-sample *t*-tests (vs. zero). A FDR correction (Benjamin and Hochberg, 1995) was applied to correct for multiple comparisons (*p* < 0.05). The key finding of this analysis was that AB magnitude was significantly different than zero in all conditions (FDR-corrected *p*’s < 0.02), except for the own name repeat condition under both low and high load (FDR-corrected *p* > 0.11). In addition, AB magnitude in other name switch condition under low load was also not significantly different than zero (FDR-correct *p* > 0.11).

### SUMMARY

The survival of personally meaningful information during the AB has been used to argue that some post-perceptual information is available during the AB (Shapiro et al., 1997). Overall performance on repeat trials replicated this previous finding showing there is no AB in response to one’s own name, but there is an AB to other people’s names. There were two main findings that were novel. First was the finding that expectancies engendered by inter-trial task dependencies modulated the severity of the AB when the second target was one’s own name. Second, overall there was an AB in both T2-own name and T2-other name conditions when T1 load switched from the previous trial. Together, both the mere presence of an AB for one’s own name on switch trials and the fact that the severity of the AB for one’s own name can be modulated by inter-trial task dependencies (i.e., AB magnitude was larger on switch relative to repeat trials) supports the idea that the post-perceptual processing of high priority stimuli can be attenuated during the AB by a violation of trial-by-trial expectancies generated during the course of one’s experience with a task. One exception to this pattern was in the other name switch condition under low load, in which the test for the presence of the AB did not reach the FDR-corrected threshold. When using an uncorrected threshold the AB magnitude was different than zero (*p* < 0.04, uncorrected), suggesting that there may be a weak AB for other names on low load switch trials. A final interesting finding is that while previous work has shown that increases in T1 task demands can cause an AB for one’s own name (Giesbrecht et al., 2009), the absence of an AB for one’s own name on repeat high load trials is suggestive evidence that the expectancies generated by inter-trial repetitions of high load are sufficient to override the effect of load on the current trial.

## DISCUSSION

The purpose of the present work was to test the extent to which expectancies about task demands engendered by the trial history of T1 task load modulate post-perceptual information processing during the AB. Experiment 1 tested the magnitude of the N400 evoked by T2 words during the AB and demonstrated that when T1 task load was repeated from the previous trial, the N400 survived the AB. Importantly, when T1 task load switched from the previous trial, the N400 evoked during the AB was attenuated relative to outside the AB. Experiment 2 tested if inter-trial dependencies influenced the extent to which personal names survive the AB. The results revealed that on T1-repeat trials one’s own name survived the AB, but other names did not. However, on T1-switch trials, an AB was present for both one’s own name and someone else’s name. This suggests that inter-trial switches of T1-load reduced the availability of highly salient information during the AB.

Previous studies have shown that manipulations of task demands within a trial can attenuate post-perceptual processing during the AB (Giesbrecht et al., 2007, 2009; Vachon et al., 2007; Vachon and Jolicoeur, 2011). The novel finding in both of the present experiments is that inter-trial dependencies of task demand, induced by repetitions and switches in T1-flanker congruency between trials, attenuated the availability of semantic information during the AB. This new finding contrasts theoretical accounts of the AB that propose that information presented during the AB is processed to a post-perceptual level despite the impairment in report (e.g., Chun and Potter, 1995; Raymond et al., 1995; Olivers and Meeter, 2008). However, the present results are consistent with the growing literature demonstrating that the failure that gives rise to the AB can occur either at post-perceptual and perceptual (i.e., pre-semantic) stages of processing (e.g., Giesbrecht et al., 2007, 2009; Vachon et al., 2007; Vul et al., 2008; Elliott and Giesbrecht, 2010). Importantly, these more recent findings suggest that the level at which selective attention operates during the AB is flexibly determined by T1-task demands (e.g., Giesbrecht et al., 2007, 2009; Vachon et al., 2007; Elliott and Giesbrecht, 2010; Vachon and Jolicoeur, 2011).

The finding that post-perceptual processing during the AB is attenuated by inter-trial dependencies of task load, parallels the finding in the visual search literature showing that post-perceptual processing of task irrelevant information is also attenuated by inter-trial switches of task demands (e.g., Theeuwes et al., 2004). These results can be explained in the context of the conflict adaptation literature that suggests that managing changes in conflict between consecutive trials is an effortful process that requires more top-down attentional control in order to resolve conflict either by an active reconfiguration of task set, or by an active inhibition of the previous task set, or both (e.g., Rogers and Monsell, 1995; Monsell, 2003; Rossi et al., 2009). However, it is important to distinguish switch costs in the traditional sense, defined by a change in stimulus-response rules, from the switch costs in the current experiments where the participants performed the identical T1 task in all trials and only the perceptual difficulty changed between trials. However, more recent work has demonstrated that perceptual switches involving changes in the number of simultaneously presented features as in the present experiments resulted in similar if not greater behavioral switch costs than when compared with more typical task-switches (cf. Ullsperger et al., 2005; Ravizza and Carter, 2008).

The availability of post-perceptual information during the AB when T1-congruency was repeated and the reduction of post-perceptual information during the AB when T1-congruency was switched between trials can be explained with a flexible selection account of attention. Flexible selection models posit that the level of information processing at which attention selects relevant information is dependent on concurrent task demands (e.g., Yantis and Johnston, 1990; Lavie, 1995, 2005; Pashler, 1998; Lavie et al., 2004; Vogel et al., 2005). In the context of the AB, an over investment of attentional resources on T1 required by a highly demanding task, such as a switch in task or high T1-load within a trial, may reduce the available resources available to process subsequent items presented rapidly beyond a perceptual level (Giesbrecht et al., 2007, 2009; Vachon et al., 2007; Elliott and Giesbrecht, 2010; Vachon and Jolicoeur, 2011). Effectively, the increase in T1-task demands increases the subsequent selectivity of processing, as measured by post-perceptual processing of T2. Thus, the present results support the proposal that the level of processing during the AB is flexible and not always fixed at a post-perceptual level and, more broadly, demonstrates that the human attention system develops expectancies about task difficulty that modulates both the spatial and temporal selectivity of attention.

## Conflict of Interest Statement

The authors declare that the research was conducted in the absence of any commercial or financial relationships that could be construed as a potential conflict of interest.

## Author Contributions

Barry Giesbrecht designed and programmed the experiments. Jocelyn L. Sy, James C. Elliott, and Barry Giesbrecht collected the data. Jocelyn L. Sy and Barry Giesbrecht analyzed the data. Jocelyn L. Sy, James C. Elliott, and Barry Giesbrecht wrote the manuscript.
